# Utilizing Mixed Cultures of Microalgae to Up-Cycle and Remove Nutrients from Dairy Wastewater

**DOI:** 10.3390/biology13080591

**Published:** 2024-08-06

**Authors:** Amira K. Hajri, Ifat Alsharif, Marzough A. Albalawi, Shareefa A. Alshareef, Raghad K. Albalawi, Bassem Jamoussi

**Affiliations:** 1 Department of Chemistry, Alwajh College, University of Tabuk, Tabuk 71421, Saudi Arabia; maalbalawi@ut.edu.sa (M.A.A.); sh.alshareef@ut.edu.sa (S.A.A.); 381007715@stu.ut.edu.sa (R.K.A.); 2 Department of Biology, Jamoum University College, Umm Al-Qura University, Makkah 21955, Saudi Arabia; eesharif@uqu.edu.sa; 3 Department of Environmental Sciences, Faculty of Meteorology, Environment and Arid Land Agriculture, King Abdulaziz University, Jeddah 21589, Saudi Arabia; bissuomaj@kau.edu.sa

**Keywords:** *Chlorella*, *Micractinium*, *Spirulina*, nutrient removal, wastewater treatment, up-cycling, biomass production, pathogen reduction

## Abstract

**Simple Summary:**

This study examines the use of dairy wastewater to cultivate three types of microalgae: *Spirulina platensis*, *Micractinium*, and *Chlorella*. The research found that these algae can effectively remove nutrients like nitrogen and phosphorus from industrial wastewater. *Spirulina* sp. and a mixed culture of all three algae performed best, significantly reducing nitrogen levels, while *Chlorella* sp. also removed a substantial amount of phosphorus. Additionally, microalgae treatment greatly reduced pathogens. These results suggest that microalgae could be a promising method for treating dairy wastewater and improving water quality.

**Abstract:**

This study explores the novel use of mixed cultures of microalgae—*Spirulina platensis*, *Micractinium*, and *Chlorella*—for nutrient removal from dairy wastewater (DW). Microalgae were isolated from a local wastewater treatment plant and cultivated under various light conditions. The results showed significant biomass production, with mixed cultures achieving the highest biomass (2.51 g/L), followed by *Spirulina* (1.98 g/L) and *Chlorella* (1.92 g/L). Supplementing DW (75%) with BG medium (25%) significantly enhanced biomass and pH levels, improving pathogenic bacteria removal. *Spirulina* and mixed cultures exhibited high nitrogen removal efficiencies of 92.56% and 93.34%, respectively, while *Chlorella* achieved 86.85% nitrogen and 83.45% phosphorus removal. Although growth rates were lower under phosphorus-limited conditions, the microalgae adapted well to real DW, which is essential for effective algal harvesting. Phosphorus removal efficiencies ranged from 69.56% to 86.67%, with mixed cultures achieving the highest removal. Microbial and coliform removal efficiencies reached 97.81%, with elevated pH levels contributing to significant reductions in fecal *E. coli* and coliform levels. These findings suggest that integrating microalgae cultivation into DW treatment systems can significantly enhance nutrient and pathogen removal, providing a sustainable solution for wastewater management.

## 1. Introduction

Daily, large amounts of wastewater are produced from various sources, and it can be treated and recycled to recover valuable nutrients like phosphorus (P), carbon (C), and nitrogen (N) [1]. The increase in pollution from water, food processing, industrial activities, and rapid economic growth threatens water quality and availability, particularly in arid and semi-arid regions. Therefore, developing effective bioremediation strategies for wastewater is important, especially from dairy production.

Dairy wastewater (DW) is characterized by high concentrations of pathogens, organic compounds, and nutrients, presenting serious environmental challenges. Inadequate management of DW can lead to significant greenhouse gas emissions, harmful health effects, and severe soil and water pollution [2]. To combat these issues, phycoremediation processes and anaerobic digestion have been utilized to improve nutrient availability and remove pollutants from DW while producing biomethane and biochemicals. This approach has succeeded in removing approximately 86% of total solids and 65.5% of chemical oxygen demand (COD). Phycoremediation uses microalgae to remove solids, reduce COD, and recover biochemicals from wastewater [3]. These processes improve water quality by breaking down organic pollutants and easing the load on conventional treatment systems. Moreover, phycoremediation supports biomethane production from algal biomass through anaerobic digestion, promoting renewable energy generation [4].

When using 25% extended digestate as the growth medium, algae can reach a biomass level of 4.64 g/L, achieving removal efficiencies of 87.1% for total phosphorus (TP), 77.6% for total nitrogen (TN), and 70.4% for COD [5]. The resulting microalgal biomass composed of approximately 8.8% lipids, 24.9% proteins, and 38.5% carbohydrates, can be co-digested with DW to produce methane (CH4) efficiently. Additionally, co-digestion with 25% algal biomass (*w*/*v*) resulted in higher methane yields (65.2%) and production rates (0.16 L/L/d) compared to other proportions [5].

Microalgae can be cultivated in wastewater using various processing strategies and membrane separation technologies [6]. For effective wastewater treatment, microalgae should exhibit high nutrient removal efficiency, biomass productivity, growth rates, and adaptability to diverse climatic and water conditions [7]. Utilizing wastewater as a nutrient source for microalgae cultivation is currently the most cost-effective method for producing microalgal biomass for biodiesel production [8].

Efforts are ongoing to develop wastewater management techniques that leverage hyper-concentrated algal cultures, which are highly efficient at removing (P) and (N) within short periods [9]. This process primarily involves nutrient uptake by the algae and inhibition of ammonia synthesis due to increased pH from photosynthesis [10,11]. Implementing nutrient removal via microalgae offers several advantages over conventional biological methods [12], including lower capital and operational costs, no need for organic carbon additives, simultaneous CO_2_ footprint reduction, and O_2_ production during treatment [13,14].

Current wastewater treatments commonly use biological and chemical methods to tackle DW, focusing on nutrient and pathogen removal. Emerging research explores microalgae due to their ability to thrive in nutrient-rich environments and effectively remove contaminants [15]. Microalgae have been applied to remediate wastewater using species such as *Chlorella vulgaris* [16], *Tribonema* sp. and *Synechocystis* sp. [17], *Spirulina platensis*, and *Scenedesmus obliquus* [18]. These techniques include using bioreactors for treating DW [19]. Also, microalgae can uptake and detoxify toxic metals, including lead, mercury, cadmium, tin, bromine, scandium, and arsenic, making them suitable for manufacturing nanoparticles and bioremediating wastewater [20,21]. Additionally, microalgae contribute to oxygen production through photosynthesis and CO_2_ fixation, supporting their growth and biomass production [22]. Furthermore, their potential to remove emerging contaminants from aquatic environments underscores their role in environmental remediation efforts.

Microalgae-based wastewater treatment technologies, such as those used by Aqualia in Chiclana, Spain, are effective in treating wastewater. Aqualia’s facility treats wastewater from 60,000 inhabitants using mixotrophic cultures of microalgae and bacteria to efficiently remove contaminants and produce clean water [23]. This method reduces land requirements and supports high biomass production. Microalgae-based processes reduce 50% of the energy consumption of conventional wastewater treatment and recover up to 90% of wastewater nutrients [24]. 

Conventional methods like electrochemical treatments, adsorption, and membrane filtration are often cost-effective [23]. Membrane technologies efficiently clean water from emerging pollutants like pharmaceuticals but lack global design standards, causing varied performance [25]. However, their integration requires adherence to specific operational criteria for efficiency across diverse conditions. In open systems for wastewater treatment, balancing high biomass productivity and growth rates involves managing variables like light exposure, temperature, and nutrient availability [26,27]. These factors are pivotal given the challenges of maintaining optimal conditions consistently, alongside addressing scalability and operational costs. 

Furthermore, harvested algal biomass can be used for anaerobic digestion to generate energy at wastewater treatment plants, with the released (N) and (P) recycled for further algae growth [28]. Municipal wastewater treatment plant secondary effluent is a persistent source of nutrient pollution, leading to eutrophication. Eutrophication, driven by excessive nitrogen (N) and phosphorus (P) inputs, accelerates algae and aquatic plant growth, depleting oxygen levels in water bodies [29]. Microalgae consortia can grow in secondary effluent, resulting in natural pH increases (approximately 9–10), effective nutrient removal, biomass production, and pathogen elimination, improving water management [30,31]. Various techniques, including biological treatments (bacteria and algae), chemical methods, anaerobic reactors, natural coagulants (such as jute processing waste and rice husk), and electro-coagulation procedures, play significant roles in DW treatment. Research has extensively investigated these methods [30,32,33,34]. However, further research is needed to optimize algae cultivation in different types of DW. 

This study aims to assess nutrient removal by microalgae in dairy wastewater treatment systems. Microalgae were isolated from different wastewater sources from primary clarifiers at a local wastewater management plant. These isolates were identified and evaluated for their growth and nutrient removal capabilities to optimize their potential applications in dairy wastewater treatment and environmental remediation. 

## 2. Materials and Methods

### 2.1. Wastewater and Dairy Wastewater Samples Used

Wastewater and dairy wastewater secondary effluent were gathered from a local wastewater treatment plant (WWTP) and pretreated by filtering through Whatman Grade 4 filter paper (pore size 20–25 µm) to remove large debris. The pretreated effluent was then stored at 4 °C and used within 24 h for isolation and cultivation experiments. *Chlorella*, *Spirulina platensis*, and *Micractinium* were grown in a modified Basal Medium tailored for the wastewater conditions, using the same container sizes and shaking methods as in the Basal Medium setup. Continuous light conditions of approximately 5000 Lux were maintained for 24 h during algae cultivation. Algal biomass was monitored by dry weight, whereas (N) and (P) concentrations were analyzed periodically throughout the cultivation period, as described by [35].

### 2.2. Isolated and Identified Microalgae

To obtain microalgae that are adaptable to dairy wastewater (DW) conditions, wastewater and dairy wastewater were collected from the WWTP in a plastic container of 10 L. The sample was cultivated in 1000 mL transparent glass bottles to isolate microalgae adapted to DW. Algal identification was carried out according to the main references used in phytoplankton identification (LUMCON’s Guide to Phytoplankton—an online taxonomic guide). *Chlorella*, *Spirulina platensis*, and *Micractinium* that became dominant were isolated and used in growth experiments with treated dairy wastewater as a culture medium. For isolation, batch sub-culturing was performed by single cells of *Chlorella*, *Spirulina platensis*, and *Micractinium*, which were picked from the culture and transferred into a 700 mL flask containing (BG-11) growth media for further cultivation for five days until exponential growth started. These algae were successively inoculated using a modified Basal Medium [36,37] to establish and maintain pure cultures of *Spirulina platensis* (blue-green algae), as well as two green algae, *Micractinium* and *Chlorella*. The inoculations were conducted at 30 °C, the optimum temperature for growth [7,38], within 1.0 L Erlenmeyer flasks containing 700 mL of working volume. Each flask was sealed with cotton wool and equipped with a gas exchange port. Cultures were kept suspended by shaking at 145 rpm using a G10 gyratory shaker. Continuous lighting conditions of 24 h (~5000 Lux) were maintained using six 40 W fluorescent lamps during algal cultivation. Fresh medium was regularly introduced to the algae cultures to sustain their purity and growth. 

### 2.3. Molecular Identification of Microalgae Isolated

This study used oligonucleotide primers to amplify 16S rDNA for *Spirulina platensis* and 18S rRNA for *Micractinium* and *Chlorella*. Following Khaw et al. [39], PCR was performed with a reaction volume of 25 μL containing PCR buffer, MgCl_2_, dNTP, primers, Pfu DNA Polymerase, and template DNA. The PCR program included initial denaturation at 94 °C for 5 min, 30 cycles of denaturation (94 °C for 1 min), annealing (55 °C for 1 min), and elongation (72 °C for 2 min), followed by a final extension at 72 °C for 10 min. The PCR product was purified and sequenced. Sequence data included *Chlorella* sp. (GenBank AB176665), *Micractinium* sp. (GenBank MH683925.1), and *Spirulina platensis* (GenBank AF527460.1), as indicated in Table 1, Table 2, Table 3 and Table 4.

### 2.4. Phylogenetic Analysis

**Figure 1:**
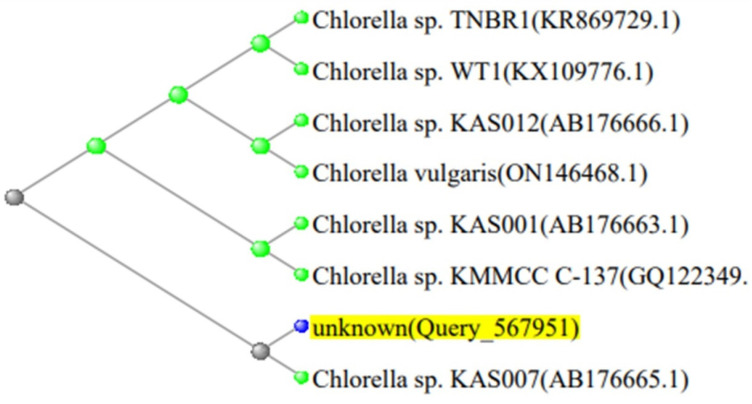
Phylogenetic tree of *Chlorella* sp. strain in contrast to the most closely related green microalgae in the NCBI database (*Chlorella* sp. AB176665.1).

**Figure 2:**
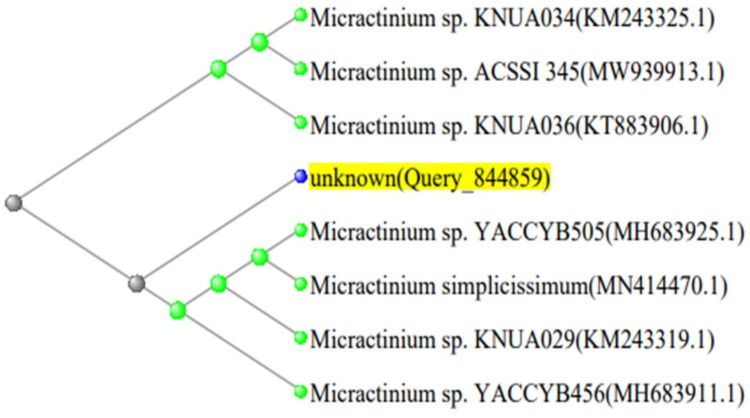
Phylogenetic tree of *Micractinium* sp. strain in contrast to the most closely related green microalgae in the NCBI database (*Micractinium* sp. MH683925.1).

**Figure 3:**
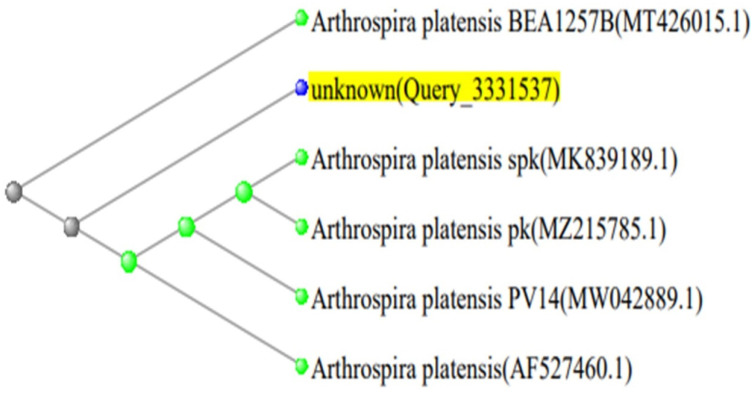
Phylogenetic tree of *Arthrospira platensis* strain in contrast to the most closely related green microalgae in the NCBI database (*Arthrospira platensis* MK839189.1).

The 16S rDNA gene sequencing of *Spirulina platensis*, and 18S rRNA gene sequencing of *Micractinium* and *Chlorella* isolates were compared to sequences in the NCBI GenBank database (http://www.ncbi.nlm.nih.gov, accessed on 25 July 2024) using the Basic Local Alignment Search Tool (BLAST). The sequence was compared to those of reference taxa found in public databases, and the evolutionary distance was calculated using NCBI Neighbor Joining. The phylogenetic trees of the three isolated strains are shown in Figure 1, Figure 2 and Figure 3.

### 2.5. Microalgae Cultivation in Real Dairy Wastewater

Microalgae were cultivated using untreated dairy wastewater secondary effluent (Table 5). *Spirulina platensis*, *Micractinium*, *Chlorella*, and a mixed culture of these three algae (in a 1:1:1 ratio) were grown in 200 mL photobioreactors. The effluent was diluted to 75% with BG-11 medium (constituents shown in Table 6). Each culture started with an inoculum size of 1.5 g/L. The microalgae were supplied with 2% CO_2_ (0.2 vvm) and maintained at 30 ± 1 °C, shaking at 200 rpm, with continuous light at an intensity of 140 μmol m^−2^ s^−1^ [40].

### 2.6. Determination of Parameters

Algae samples were centrifuged for 5 min at 6000 rpm, and then suspended solids were filtered from the supernatant using a 0.45 μm cellulose membrane [39]. Samples were collected throughout algal growth to measure biomass and dairy wastewater performance (TP and TN levels). Microalgal cultivation was conducted in triplicate. The difference between the initial and final factors was calculated using the formula (A − B)/B × 100, where A is the initial level and B is the level after growth during analysis intervals. The negative control of all experiments was conducted in the same environmental conditions without algae inoculation, and no significant change was recorded in the results throughout the incubation period.

The total harvested biomass was oven-dried at 70 °C for 48 h following AOAC [41]. Phosphorus and nitrogen levels were determined using a spectrophotometer (U-5100, Hitachi, Tokyo, Japan) [35], and pH was measured with a pH meter [42]. Algal biomass was assessed at 680 nm optical density (OD_680_) using a UV/Vis spectrophotometer (U-5100, Japan) [28]. 

### 2.7. Determination of Bacterial Indicator for the Presence of Pathogens

Wastewater samples (10 mL) were transferred into a sterile Erlenmeyer flask and diluted with 90 mL of sterile buffered peptone water (1 g/L peptone, pH 7.2). Serial decimal dilutions were then made to 10^−8^ using the buffer solution. Total bacterial counts (TBCs) for wastewater samples were performed in triplicate according to the American Public Health Association (APHA, 2012) guidelines. Samples were plated on plate count agar and incubated at 30 °C for 48 h. Results are reported as average (Log_10_) with documented standard error (SE).

Then, 1.0 mL from the dilution sample was added to a sterile Petri dish with 10 mL of Violet Red Bile Dextrose Agar (Biolife, Italy) for the total coliform count. After solidifying, another 10 mL of the same melted media was added, and the culture was incubated at 37 °C for 24 h. Total coliforms were determined using the Hach method 8001 and expressed as the most probable number (MPN). Following the manufacturer’s instructions, Escherichia coli was identified using ChromoCult Coliform agar (Merck KGaA, Berlin, Germany). The incubation was performed at 44 °C for 24 h and confirmed with Kovac’s indole reagent. The difference between initial and final numbers was calculated using the formula (A − B)/B × 100, where A is the initial number and B is the count after microalgae growth during analysis intervals for calculating the removal efficiency (RE) of pathogens.

### 2.8. Statistical Analysis

Data were analyzed using one-way analysis of variance (ANOVA) with SPSS Statistics 24.0 (IBM, New York, NY, USA). A *p*-value of 0.05 was used to determine significant differences between treatments.

## 3. Results

### 3.1. Cultivation of Microalgae in Real Dairy Wastewater

**Figure 4:**
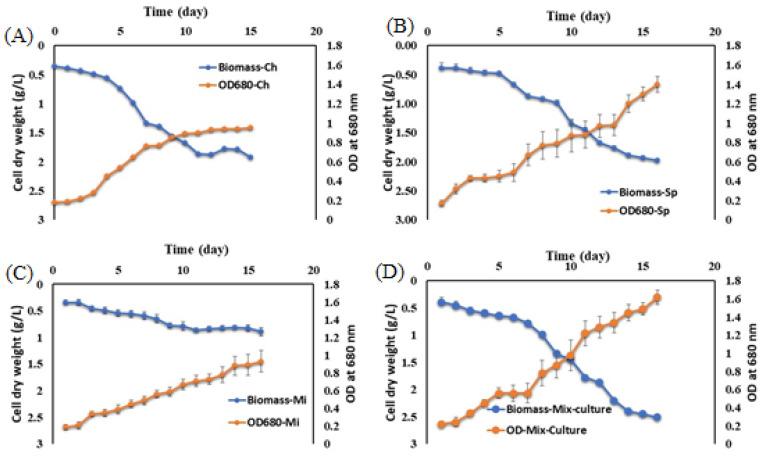
Microalgae grown (cell dry weight (g/L) and OD at 680 nm) of (**A**) *Chlorella* sp., (**B**) *Spirulina* sp., (**C**) *Micractinium* sp., and (**D**) mixed culture of three microalgae in the primary effluent of dairy wastewater.

Based on the isolation results, the morphological characteristics of the isolated algae showed that they were *Spirulina* sp., *Micractinium* sp., and *Chlorella* sp. Figure 4 shows the biomass dry weight and optical density (OD) at 680 nm during cultivation. All species grew well in the secondary effluent, with the highest biomass achieved in a mixed culture (2.51 g/L), followed by *Spirulina* and *Chlorella* (1.98 and 1.92 g/L, respectively) in the real DW effluent. *Micractinium* had the lowest biomass and OD at 680 nm. In DW secondary effluent media, Spirulina and the mixed culture showed the highest OD at 680 nm (1.40 and 1.62, respectively).

The biomass of the three microalgae increased gradually at 30 °C over 15 days, with OD_680_ rising concomitantly. At the end of cultivation growth, biomass and OD_680_ levels reached a comparatively higher maximum value. Supplementing DW with BG medium (25%) significantly improved biomass. In addition, supplementing DW with the medium significantly increased the pH value (*p* < 0.05). This rise in pH contributed to a higher efficiency in eliminating pathogenic bacteria. Chlorella and Spirulina’s average specific growth rate ranged between 0.1 and 0.21 d^−1^.

### 3.2. Effect of pH in Dairy Wastewater Effluent and Microalgae Biomass Production

**Figure 5:**
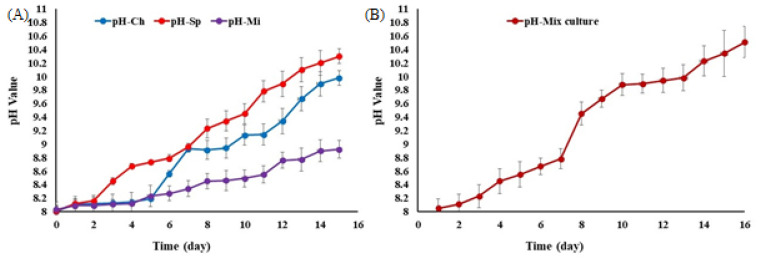
Changes in pH value of the real medium of dairy wastewater during microalgae cultivation.

Using dairy wastewater as a secondary effluent medium for microalgae cultivation significantly improved the pH value (*p* < 0.05), reaching its highest level after 15 days of cultivation. At the end of the experiment, the initial pH of approximately 8.0 increased for all cultures: *Micractinium* reached 8.92, *Chlorella* reached 9.98, *Spirulina* reached 10.30, and the mixed cultures reached 10.51 (Figure 5).

### 3.3. Nitrogen and Phosphorous Removal during Algae Cultivation

**Figure 6:**
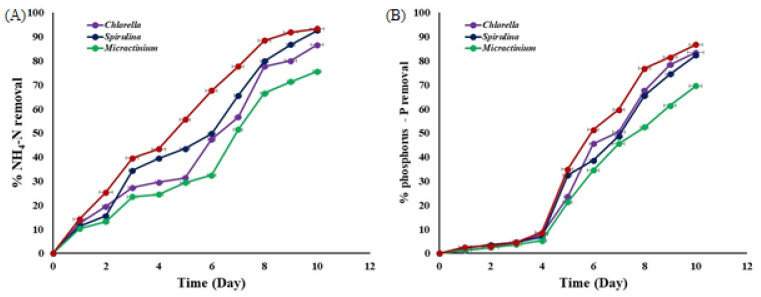
Total % NH_4_-N removal and % phosphorus—P removal during cultivation of microalgae in dairy wastewater.

The nitrogen removal efficiency for microalgae grown in DW secondary effluent ranged from 75.86% to 93.34% (Figure 6). Under 24 h light conditions and 75% DW mixed with 25% BG medium, *Spirulina* and the mixed culture showed the highest N removal efficiencies at 92.56% and 93.34%, respectively. In comparison, *Chlorella* achieved 86.85% N removal and *Micractinium* achieved 75.56%.

The phosphorus removal efficiency ranged between 69.56% and 86.67% for the cultivation of the DW secondary effluent (Figure 6). The mixed culture of the three algae removed 86.67% of soluble P under light conditions. Individually, each culture’s P removal efficiency ranged from 69.56% to 83.45%. Fewer than 24 h light conditions, Chlorella had the highest P removal at 83.45%, while *Spirulina* had a removal efficiency of 83.34%. *Micractinium* had the lowest P removal efficiency under the same conditions. Overall, the mixed cultures removed 87% of soluble phosphorus.

### 3.4. Microbial and Coliform Removal during Algal Cultivation

The total bacterial count (TBC) and the removal efficiency for pathogenic bacteria (coliform and *E. coli*) in dairy wastewater reached between 88.33% and 97.81% (Table 7, Table 8 and Table 9). The decline in coliform bacterial groups and fecal *E. coli* levels was significant through various wastewater treatment methods. In this study, the improved pH of the dairy wastewater secondary effluent provided hostile conditions for fecal *E. coli* and total coliforms, reducing their levels to less than 1.0 Log_10_ CFU/mL and achieving a removal efficiency of 97.81% (Table 9).

## 4. Discussion

The study demonstrated that microalgae, including *Chlorella*, *Spirulina*, and *Micractinium*, can be effectively cultivated in real dairy wastewater. The mixed culture showed the highest biomass and optical density, indicating better adaptation to harsh conditions [16,17,18]. The addition of BG medium significantly enhanced biomass growth and pH, improving the removal of pathogenic bacteria [33].

### 4.1. Cultivation of Microalgae in Real Dairy Wastewater

Microalgal genera isolated from wastewater included *Chlorella*, *Spirulina*, and *Micractinium*. Specifically, *Chlorella vulgaris*, *Tribonema* sp., *Synechocystis* sp., *Spirulina platensis*, and *Scenedesmus obliquus* have been used for bioremediation of polluted water [17,18]. However, microalgal wastewater management requires significant land space. Morphological analysis of microalgae from a local WWTP identified three species: *Chlorella* sp., *Spirulina* sp., and *Micractinium* sp. The results indicated that mixed cultures, *Spirulina*, and *Chlorella* sp., were more tolerant of the harsh conditions in dairy wastewater than *Micractinium*. 

The data showed that DW secondary effluent is an effective medium, promoting growth when supplemented with synthetic medium and longer light exposure. Algae grown under these conditions exhibited relatively higher specific growth rates for all species. For example, the specific growth rates of *Chlorella* sp. in sludge centrate ranged from 0.19 to 0.68 d^−1^ [43]. Our results showed lower specific growth rates than other studies, suggesting that the availability of carbon dioxide, light, and nutrients is a limiting factor for algae growth. This observation aligns with findings from Maleki Samani and Mansouri [33], who found that DW can serve as a suitable culture medium for *Chlorella vulgaris*. Sufficient nutrients, such as N and P, enhance microalgae growth and performance in DW environments [44,45,46]. Our study supports the use of microalgae to remove nutrients from dairy wastewater and offers an economical method for treating industrial wastewater and producing biomass for energy production [34,47]. This dual benefit underscores the potential of microalgae-based systems in sustainable wastewater management practices. Studies show that microalgae used in DW treatment reduce chemical use and generate valuable by-products like biofuels and fertilizers [48]. Our study supports these findings by demonstrating the dual benefits of effective wastewater treatment and valuable by-product generation, contributing to a circular bioeconomy.

### 4.2. Effect of pH in Dairy Wastewater Effluent and Microalgae Biomass Production

The current study leveraged the natural increase in pH as a key operational variable, maintaining pH values ≤ 10. pH levels exceeding 10.33 promote the prevalence of CO_3_^2−^, which inhibits carbon uptake and microalgae growth [49]. Changes in pH during microalgae cultivation are primarily regulated by suspended inorganic carbon species (H_2_CO_3_, HCO^3−^), which act as pH buffers [50]. HCO^3−^ is identified as the predominant form of dissolved inorganic carbon in aqueous environments, with a pH ranging from 6.36 to 10.33 [11,51], highlighting the importance of maintaining these pH levels for optimal conditions. Our study offers new insights into optimizing microalgae cultivation conditions specific to dairy wastewater, thereby contributing to the refinement of bioprocesses in this field.

In our study, mixed cultures demonstrated superior growth compared to monocultures. This improvement is due to complementary metabolic interactions, enabling more efficient nutrient use [52,53]. The diversity within mixed cultures enabled better exploitation of various nutrient forms, enhancing overall productivity [54]. Their resilience to environmental stressors also led to more stable growth and efficient nutrient removal [55].

### 4.3. Nitrogen and Phosphorous Removal during Algae Cultivation

Microalgae can adapt their physiological pathways to challenging environmental conditions such as wastewater, high temperatures, or extreme salinity [56]. Their growth depends on light exposure, nutrient availability, and temperature [57]. The composition of microalgae biomass is influenced by growth conditions such as temperature, pH, light intensity, and nutrient availability [58]. Considering these factors is crucial for optimizing growth conditions.

Different types of microalgae respond differently to their environments, affecting their productivity and growth [6]. For example, *Chlorella vulgaris* shows higher lipid productivity at lower temperatures, while optimal growth occurs at 30 °C [38].

Characteristic wastewater contains diverse inorganic constituents including calcium, phosphates, ammonium salts, magnesium, sodium, potassium, bicarbonates, chlorine, sulfur, and toxic metals. Additionally, organic carbon is present in fats, volatile acids, proteins, amino acids, and carbohydrates. Levels of P and N are critical for microalgae development, influencing their growth kinetics and nutrient uptake, crucial for nutrient removal and biomass accumulation [57]. The production of biomass is crucial. Recent research shows that microalgae like *Scenedesmus obliquus* can be cultivated in dairy effluent to produce valuable biomass for biofuels and biostimulants [48]. Our results contribute to this body of knowledge by confirming high biomass yields and exploring the potential for biofuel production from the harvested biomass.

*Chlorella thermophila* has shown promise in recovering nutrients from dairy wastewater, potentially remedying wastewater by utilizing its nutrients and offering plant protection against harmful phytopathogens [59]. Recent studies have highlighted the efficiency of various microalgae species in removing nutrients from dairy wastewater. For instance, *Chlorella vulgaris* has shown significant capabilities in removing nitrogen and phosphorus, achieving removal rates up to 90% and 85%, respectively [34]. Additionally, increasing *C. vulgaris* concentration from 1 to 10 g/L enhanced removal rates: TN from 81.04% to 84.81%, TP from 32.26% to 36.12%, NH_3_-N from 96.90% to 97.26%, and PO_4_-P from 44.76% to 48.71% [60]. Moreover, Gupta et al. [61] achieved 95% TN removal, in addition to phosphorus removal efficiencies exceeding 68% and 99% NH_3_–N using mixed microalgae cultures in DW. Comparatively, our study also demonstrates high nutrient removal efficiencies, aligning with these recent findings and confirming the effectiveness of microalgae in nutrient uptake from DW.

Due to several key factors, mixed cultures of microalgae outperform monocultures in removing (N) and (P) from DW. Firstly, the synergy among different species enhances nutrient removal, as they can release enzymes or compounds that make nutrients more accessible to others within the consortium [34,62]. Secondly, diverse species in mixed cultures have varying mechanisms for nutrient uptake, allowing them to absorb a broader range of nitrogen and phosphorus forms, thus improving overall efficiency [54,63]. Additionally, mixed cultures offer greater resilience to environmental fluctuations, such as changes in temperature and pH, ensuring stable nutrient removal even under less-than-ideal conditions [64]. Lastly, forming biofilms or aggregates by certain species in mixed cultures enhances nutrient retention and uptake, further boosting removal processes [65].

Studies have integrated microalgae cultivation with systems like activated sludge or co-cultivation with fungi to enhance treatment efficiency and biomass production [34,66]. Our research compares standalone microalgae efficiency with these systems, highlighting their benefits and limitations. Our results reinforce the current understanding of microalgae-based DW treatment by providing contemporary data on nutrient removal efficiencies, biomass production rates, and potential by-product applications.

### 4.4. Microbial and Coliform Removal during Algal Cultivation

Microalgae cultivation has demonstrated significant potential in removing microbial contaminants such as *E. coli* from domestic wastewater. Studies have shown that pH levels between 8 and 9.5 can reduce the *E. coli* population by approximately 50% and 100%, respectively [67]. Microalgae are also effective in removing N, P, and other nutrients from various types of wastewater, often elevating the pH of the medium to around 10.0 [11,12,43,68], as shown in (Figure 2). 

In secondary effluent from wastewater treatment plants, microalgae consortia naturally elevate pH levels to approximately 9–10. This natural pH increase enhances nutrient removal and promotes the growth of microalgal biomass, which benefits from the bioavailability and preservation of (P) and (C). This biomass production not only aids in pathogen elimination but also supports effective water management strategies [30,31]. 

Microalgae produce reactive oxygen species (ROS) under light exposure, which are lethal to bacteria, enhancing pathogen removal in wastewater [69]. Our research shows that combining photoinactivation with increased pH creates hostile conditions for bacteria, significantly reducing their populations. While pH is crucial, other factors enhance microalgae’s effectiveness in dairy wastewater treatment. Microalgae like *Chlorella* and *Spirulina* produce antimicrobial peptides that inhibit bacterial growth [70,71]. Additionally, mixed cultures produce a broader range of antimicrobial compounds, further improving treatment efficacy [65]. Numerous studies have explored the use of microalgae for treating domestic wastewater [30,32,33]. The combined effects of pH elevation and nutrient removal significantly reduce bacterial populations in the medium.

## 5. Conclusions

This study demonstrates the potential of using dairy wastewater (DW) for cultivating microalgae, specifically *Spirulina platensis*, *Micractinium*, and *Chlorella*, to enhance wastewater treatment. The microalgae showed robust growth and significant nutrient removal, with mixed cultures achieving the highest biomass yields. Maintaining pH levels around 9 to 10 during microalgae cultivation created unfavorable conditions for *E. coli* and coliforms, while preserving phosphorus and carbon bioavailability. The adaptability of microalgae to varying DW conditions underscores their suitability for large-scale applications, offering a sustainable, cost-effective method for nutrient removal, biomass production, and improved wastewater management. Future studies should focus on optimizing cultivation conditions and exploring the commercial viability of large-scale applications.

## Figures and Tables

**Table 1:** 16S rDNA and 18S rDNA oligonucleotide primers used in the current study.

Primer	Sequencing (5–3′)	Primer Name	Position	Purity Degree
Forward	CCTGGAAGGGGCGTATTTAT	Univ. contig,16S rRNA gene, RS_001101.5	*Arthrospira platensis* (*Spirulina platensis*)	98%
Reverse	CTTGGATGTGGTAGCCGTTT			
Forward	CGACTAGCCAATGGAAGCAT	Univ. contig, 18S rRNA gene, GR_001142797.2	*Chlorella* sp. *Micractinium* sp.	98%
Reverse	GTACAAAGGGCAGGGACGTA			

**Table 2:** GenBank accession numbers, closest phylogenetic relative, and identity percent of the *Chlorella* strain.

Description	Query Cover	Strain	Identity %	Accession
*Chlorella* sp. KAS007 gene for 18S rRNA gene.	100%	*Chlorella* sp.	100.00	AB176665.1
*Chlorella* sp. TNBR1 18S ribosomal RNA gene.	100%		99.94	KR869729.1
*Chlorella* sp. KAS012 gene for 18S rRNA gene.	100%		99.94	AB176666.1
*Chlorella* sp. KAS001 gene for 18s rRNA gene.	100%		99.94	AB176663.1
*Chlorella* sp. WT1 18S ribosomal RNA gene.	100%		99.88	KX109776.1
*Chlorella vulgaris* isolate BEA 0046B small subunit ribosomal RNA gene and internal transcribed spacer 1.	100%		99.88	ON146468.1
*Chlorella* sp. KMMCC C-137 18S ribosomal RNA gene.	100%		99.88	GQ122349.1

**Table 3:** GenBank accession numbers, closest phylogenetic relative, and identity percent of the *Micractinium* strain.

Description	Query Cover	Strain	Identity %	Accession
*Micractinium* sp. YACCYB505 18S ribosomal RNA gene.	100%	*Micractinium* sp.	100.00	MH683925.1
*Micractinium* sp. KNUA029 18S ribosomal RNA gene.	100%		100.00	KM243319.1
*Micractinium* sp. YACCYB456 18S ribosomal RNA gene.	100%		99.94	MH683911.1
*Micractinium* sp. KNUA034 18S ribosomal RNA gene.	100%		99.94	KM243325.1
*Micractinium* sp. KNUA036 18S ribosomal RNA gene.	100%		99.88	KT883906.1

**Table 4:** GenBank accession numbers, closest phylogenetic relative, and identity percent of the *Arthrospira platensis* (*Spirulina platensis*) strain.

Description	Query Cover	Stain	Identity %	Accession
*Arthrospira platensis* spk 16S ribosomal RNA gene.	100%	*Arthrospira platensis*	100.00	MK839189.1
*Arthrospira platensis* pk 16S ribosomal RNA gene.	100%	*Arthrospira platensis*	100.00	MZ215785.1
*Arthrospira platensis* PV14 16S ribosomal RNA gene.	100%	*Arthrospira platensis*	100.00	MW042889.1
*Spirulina platensis* 16S ribosomal RNA gene.	100%	*Spirulina platensis*	100.00	AF527460.1
*Arthrospira platensis* BEA1257B 16S ribosomal RNA gene.	100%	*Arthrospira platensis*	99.48	MT426015.1

**Table 5:** Medium characteristics used for microalgae cultivation with dairy wastewater (DW) secondary effluent.

Parameter	Value
COD	1180 ± 0.78 mg/L
pH	8.11 ± 0.14
EC	450.59 ± 0.57 mS/m
Nitrate nitrogen (NO_3_^−^-N)	19.45 ± 0.47 mg/L
Phosphate phosphorus (PO_4_^3−^-P)	3.83 ± 0.56 mg/L
Total alkalinity	308.67 ± 0.87 mg/L as CaCO_3_
TBC	6.74 ± 0.45 Log_10_ CFU/mL
Total coliform	17.56 ± 0.35 MPN/100 m
*Escherichia coli*	15.67± MPN/100 mL

Mean (n = 10) ± standard error (SE). Chemical oxygen demand (COD); electrical conductivity (EC); total bacterial count (TBC); most probable number (MPN).

**Table 6:** The composition of BG-11 medium used in this experiment.

Elements	Amount (g/L)	Elements	Amount (g/L)
NaNO_3_	1.5	H_3_BO_3_	2.8 × 10^−3^
MgSO_4_·7H_2_O	0.075	MnCl_2_·4H_2_O	1.8 × 10^−3^
K_2_HPO_4_	0.04	Na_2_MoO_4_·2H_2_O	3.9 × 10^−4^
CaCl_2_	0.036	ZnSO_4_·7H_2_O	2.2 × 10^−4^
Na_2_CO_3_	0.02	CuSO_4_·5H_2_O	7.9 × 10^−5^
Citric acid	0.006	Co(NO_3_)_2_·6H_2_O	4.9 × 10^−5^
Ferric (ammonium) citrate	0.006	EDTA·2Na	0.001

**Table 7:** Removal efficiency of total bacterial count (% percentage ± standard deviation) by cultivation of microalgae in dairy wastewater.

Time (day)	*Chlorella*	*Spirulina*	*Micractinium*	Mixed Cultures
0	0	0	0	0
1	5.84 ± 0.19 ^j^	5.53 ± 0.12 ^k^	3.93 ± 0.43 ^lm^	6.17 ± 0.58 ^k^
2	6.15 ± 0.41 ^j^	5.71 ± 0.26 ^k^	4.85 ± 0.51 ^l^	7.79 ± 0.62 ^k^
3	10.12 ± 0.35 ^i^	6.78 ± 0.24 ^k^	5.69 ± 0.45 ^l^	12.15 ± 0.86 ^j^
4	12.31 ± 0.33 ^h^	9.66 ± 0.32 ^j^	8.81 ± 0.50 ^k^	14.15 ± 0.89 ^i^
5	25.11 ± 0.24 ^g^	17.78 ± 0.13 ^h^	14.61 ± 0.20 ^j^	26.64 ± 0.90 ^h^
6	45.61 ± 0.12 ^f^	51.56 ± 0.15 ^g^	46.53 ± 0.21 ^i^	52.39 ± 0.25 ^g^
7	73.81 ± 0.12 ^e^	70.78 ± 0.14 ^f^	54.54 ± 0.22 ^h^	78.72 ± 0.44 ^f^
8	81.62 ± 0.22 ^d^	74.67 ± 0.23 ^be^	63.61 ± 0.21 ^g^	83.71 ± 0.54 ^e^
9	83.51 ± 0.14 ^c^	77.67 ± 0.21 ^d^	68.18 ± 0.23 ^f^	86.48 ± 0.56 ^d^
10	86.54 ± 0.16 ^bc^	78.95 ± 0.17 ^d^	74.38 ± 0.19 ^e^	89.37 ± 0.63 ^c^
15	87.17 ± 0.13 ^bc^	81.91 ± 0.19 ^c^	79.55 ± 0.13 ^d^	90.27 ± 0.44 ^c^
20	89.18 ± 0.13 ^b^	82.33 ± 0.15 ^c^	81.57 ± 0.16 ^c^	93.47 ± 0.19 ^b^
25	91.71 ± 0.12 ^b^	84.65 ± 0.19 ^b^	87.23 ± 0.27 ^b^	94.11 ± 0.24 ^b^
30	93.51 ± 0.16 ^a^	87.83 ± 0.18 ^a^	90.34 ± 0.23 ^a^	94.41 ± 0.21 ^b^
35	94.15 ± 0.14 ^a^	88.55 ± 0.14 ^a^	91.47 ± 0.21 ^a^	96.14 ± 0.22 ^a^
40	95.16 ± 0.18 ^a^	88.33 ± 0.21 ^a^	91.11 ± 0.22 ^a^	97.21 ± 0.43 ^a^

Means in the same column with different superscript letter following them are significantly different (*p* < 0.05).

**Table 8:** Removal efficiency of coliform (% percentage ± standard deviation) by cultivation of microalgae in dairy wastewater.

Time (day)	*Chlorella*	*Spirulina*	*Micractinium*	Mixed Cultures
0	0	0	0	0
1	2.34 ± 0.13 ^i^	4.53 ± 0.12 ^h^	3.53 ± 0.23 ^h^	5.47 ± 0.18 ^g^
2	3.45 ± 0.11 ^h^	5.71 ± 0.16 ^g^	3.85 ± 0.21 ^h^	6.73 ± 0.12 ^g^
3	5.65 ± 0.12 ^g^	6.78 ± 0.14 ^g^	4.65 ± 0.15 ^g^	8.65 ± 0.16 ^f^
4	8.36 ± 0.23 ^f^	5.66 ± 0.12 ^g^	4.86 ± 0.21 ^h^	8.94 ± 0.14 ^f^
5	9.31 ± 0.14 ^f^	7.78 ± 0.13 ^f^	5.67 ± 0.27 ^g^	9.64 ± 0.10 ^f^
6	56.67 ± 0.16 ^e^	51.56 ± 0.15 ^e^	43.53 ± 0.26 ^e^	59.34 ± 0.15 ^e^
7	78.84 ± 0.15 ^d^	77.78 ± 0.14 ^d^	55.56 ± 0.12 ^d^	83.79 ± 0.14 ^d^
8	89.67 ± 0.12 ^c^	86.67 ± 0.13 b^c^	66.67 ± 0.11 ^c^	89.69 ± 0.24 ^c^
9	95.56 ± 0.13 ^b^	88.67 ± 0.11 ^b^	69.58 ± 0.13 ^c^	96.68 ± 0.16 ^b^
10	94.56 ± 0.11 ^b^	89.9 ± 0.07 ^b^	73.78 ± 0.17 ^c^	95.87 ± 0.33 ^b^
15	94.87 ± 0.17 ^b^	89.97 ± 0.09 ^b^	88.45 ± 0.15 ^bc^	95.67 ± 0.14 ^b^
20	94.98 ± 0.17 ^b^	90.34 ± 0.11 ^ab^	89.67 ± 0.12 ^bc^	97.45 ± 0.13 ^ab^
25	95.78 ± 0.15 ^b^	90.67 ± 0.18 ^ab^	90.23 ± 0.27 ^ab^	97.34 ± 0.14 ^ab^
30	96.56 ± 0.13 ^ab^	90.88 ± 0.15 ^ab^	91.34 ± 0.23 ^ab^	97.45 ± 0.11 ^a^
35	97.45 ± 0.19 ^a^	93.56 ± 0.12 ^a^	92.47 ± 0.21 ^a^	98.12 ± 0.12 ^a^
40	97.56 ± 0.12 ^a^	94.34 ± 0.23 ^a^	93.11 ± 0.22 ^a^	98.23 ± 0.23 ^a^

Means in the same column with different superscript letter following them are significantly different (*p* < 0.05).

**Table 9:** Removal efficiency of *Escherichia coli* (% percentage ± standard deviation) by cultivation of microalgae in dairy wastewater.

Time (day)	*Chlorella*	*Spirulina*	*Micractinium*	Mixed Cultures
0	0	0	0	0
1	1.31 ± 0.33 ^l^	1.58 ± 0.42 ^j^	1.51 ± 0.53 ^j^	3.31 ± 0.16 ^m^
2	1.41 ± 0.31 ^l^	1.76 ± 0.56 ^j^	1.45 ± 0.41 ^j^	7.78 ± 0.22 ^gl^
3	1.64 ± 0.22 ^l^	1.73 ± 0.24 ^j^	1.75 ± 0.35 ^j^	10.51 ± 0.46 ^f^
4	2.37 ± 0.53 ^k^	1.62 ± 0.32 ^j^	1.83 ± 0.61 ^j^	18.18 ± 0.54 ^k^
5	7.32 ± 0.24 ^j^	1.78 ± 0.33 ^j^	1.69 ± 0.37 ^j^	29.44 ± 0.60 ^j^
6	17.69 ± 0.26 ^i^	14.53 ± 0.55 ^i^	13.53 ± 0.21 ^i^	41.75 ± 0.85 ^i^
7	28.86 ± 0.25 ^h^	17.73 ± 0.24 ^h^	15.66 ± 0.22 ^h^	53.79 ± 0.16 ^h^
8	55.17 ± 0.22 ^g^	46.91 ± 0.23 ^g^	46.69 ± 0.31 ^g^	69.69 ± 0.22 ^g^
9	67.53 ± 0.23 ^f^	58.67 ± 0.31 ^f^	55.51 ± 0.33 ^f^	76.34 ± 0.36 ^f^
10	77.56 ± 0.21 ^de^	71.49 ± 0.17 ^e^	70.18 ± 0.27 ^e^	85.17 ± 0.43 ^e^
15	79.87 ± 0.27 ^d^	79.17 ± 0.39 ^d^	71.15 ± 0.19 ^e^	88.76 ± 0.24 ^cd^
20	89.91 ± 0.27 ^c^	83.31 ± 0.31 ^c^	79.17 ± 0.18 ^d^	91.75 ± 0.33 ^c^
25	91.71 ± 0.25 ^c^	88.64 ± 0.28 ^b^	85.21 ± 0.23 ^c^	95.97 ± 0.44 ^b^
30	94.55 ± 0.23 ^b^	89.83 ± 0.25 ^b^	88.35 ± 0.24 ^b^	96.96 ± 0.51 ^a^
35	95.34 ± 0.13 ^b^	91.51 ± 0.32 ^a^	90.48 ± 0.24 ^a^	97.95 ± 0.32 ^a^
40	98.59 ± 0.11 ^a^	92.31 ± 0.13 ^a^	91.19 ± 0.21 ^a^	97.81 ± 0.63 ^a^

Means in the same column with different superscript letter following them are significantly different (*p* < 0.05).

## Data Availability

All data presented in this article are available within the manuscript. Any additional data or inquiries can be obtained from the corresponding author upon request.
